# The impact of the COVID-19 pandemic upon pancreatic cancer treatment (CONTACT Study): a UK national observational cohort study

**DOI:** 10.1038/s41416-023-02220-2

**Published:** 2023-03-23

**Authors:** Lewis A. Hall, Siobhan C. McKay, James Halle-Smith, Joshua Soane, Daniel-Clement Osei-Bordom, Lesley Goodburn, Laura Magill, Thomas Pinkney, Ganesh Radhakrishna, Juan W. Valle, Pippa Corrie, Keith J. Roberts, The CONTACT Study Group, Lesley Goodburn, Terry Hughes, Rita Perry, Michael Walters, Radhika Acharya, Thomas Binnersly, Samuel J. Brown, Rebecca Everitt, Oscar Hargreaves, Samuel Hodgson, Jacek Parylo, Madeleine Perrett, Daniel J. Smith, Thomas Thorne, Mohamed Abouelazayem, Alison Bradley, Chelise Currow, Richard Fox, Benjamin Giles, Amar Kourdouli, Fahad Mahmood, John Moir, Nicholas Mowbray, Rohan Shotton, Cavitha Vivekananthan, Roxanna Zakeri, Reyad Abbadi, Sian Abbott, Hamza Abdelrahim, Yusria Abukar, Nader Adel, Hussamuddin Adwan, Marriam Ahmed, Shehzad Ahmed, Irfan Ahmed, Ouiam Akotat, Bilal Al-Sarireh, Amro Alamassi, Gemma Aldous, Bassam Alkari, Ahmed Almonib, Jasim Amin, Muhammad Raheel Anjum, Somaiah Aroori, Ali Arshad, Pallavi Arya, Syed Asfandyar, Usama Aslam, Richard Aspinall, Tejinderjit Athwal, Saima Azam, Robert Bailey, Nanda Bandlamudi, Sophie Barker, Khalid Bashir, Akshay Bavikatte, Raluca Belchita, Ann Beluso, Katharine Bevan, Imran Bhatti, Amal Boulbadaoui, Tamsin Boyce, Neil Bradley, Corinne Brooks, Christopher Brown, Stephanie Burns, Linda Butler, Hannah Byrne, Ruben Canelo, Carlo Ceresa, Georgina Chadwick, Irene Charlesworth, Thomas Chase, Patrick Chen, Raunaq Chhabra, Mei Ying Chin, Zeshan Choudhry, Yooyun Chung, Svetlana Ciocarlan, Jennifer Clark, Danielle Clyde, Maureen Connolly, Kathleen Connors, Jonathan Cormack, Meghan Coyle, Andrew Crumley, Nick Davies, Emma Davies, Nicola de Liguori-Carino, Filippo Di Franco, Kok Diong, Matt Doe, Victoria Donovan, Jennifer Downs, Trish Easton, Tolu Ekong, Utitofon Ekpenyong, Tarek El-housseri, Ahmed Elmaradny, Mohamed Elzubier, Emmanouil Epanomeritakis, Marios Erotocritou, Iain Ewing, Christiana Fabelurin, Stephen Falk, Alexia Farrugia, Michael Feretis, Guy Finch, Alasdair Findlay, Simon Fisher, Steven Fong, Katherine Fox, Xavier Fung, Giuseppe Fusai, Laura Gale, Tamara Gall, Giuseppe Garcea, Jaber Gasem, Fanourios Georgiades, Joe Geraghty, Nader Ghassemi, Mustafa Gherghab, Joanne Giles, Roopinder Gillmore, Leah Gilroy, Matthew Goldsworthy, Alex Grayston, Jordan Green, Roy Gurprashad, Wafaa Hajee-Adam, Shahin Hajibandeh, Clara Hallinan-Rhodes, Adel Hamed, Waseem Hameed, Siddartha Handa, Michael Hanna, Mohammad Hassan, Tim Havard, Jennifer Hayes, Philip Hayton, Madhu Hebbar, Kerri-Marie Heenan, Christine Higgins, Michael Ho, David Holroyd, Richard Howard, Charlotte Hughes, Nashiz Inayet, Sahra Indayare, Julie Ingmire, Glen Irving, Anita Ivimy, Georgina Jackson, Asif Jah, Nigel Jamieson, Shameen Jaunoo, Nasir Javed, Arun Jeevagan, Long Jiao, Sarah Johnson, Miriam Jones, Michael Jones, Claire Jones, Dylan Jones, Vicky Jones, Caitlin Jordan, Paul Jose, Neerav Joshi, Kunal Joshi, Daniel Kane, Diya Kapila, Syed Karim, Muthi Kasimanickam, Mandeep Kaur, Ambareen Kausar, Ben Keatley, Adam Kedzierski, Deepak Kejariwal, Natasha Kelly, Areeb Khan, Aria Khani, Usman Khokar, William Knibbs, Hemant Kocher, Ioannis Koumoutsos, Shemin Kovammal, Sarah Kreppel, Tharsika Kuganesan, Yogesh Kumar, Reuben Kurien, Nikhil Lal, Corina Lavelle, Sophie Laverick, Lauren Laverty, Hemant Laxaman, Alvin Lee, Karen Lloyd-Jones, Pavlos Lykoudis, Aarini Mahalingam, Agata Majkowska, Debasis Majumdar, Yogeshkumar Malam, Kulbir Mann, Robyn Marsh, Harry Martin, Joseph Mcaleer, Stephen McCain, Hannah McCaughan, Catherine McCollum, Kieran McCormack, Claire McDonald, John McGoran, Morag Mclellan, Joseph Meilak, Shyam Menon, Donald Menzies, James Milburn, Andrew Millar, Moeed Minto, Amitabh Mishra, Zain Mitha, Vikramjit Mitra, Sathis Mogan, Badreldin Mohamed, Ghazaleh Mohammadi-Zaniani, Yaser Mohammed, Jaiganesh Mohan, Samuel Morris, Gary Morrison, Tamsin Morrison, Matthew Mortimer, Samuel Moulding, Moustafa Mourad, Sujit Mukherjee, Ameer Mustafa, Keval Naik, Syed Naqvi, Deepika Natarajan, Thomas Ngan, Tracey Noakes, Tim Norris, Elizabeth O’Connell, Rebecca O’Kane, David O’Reilly, William O’Rourke, Olaolu Olabintan, Samuel Ololade, Seok Ling Ong, Oluwafemi Osunlusi, Altaf Palejwala, Anna Palepa, Monica Palmer, Constantinos Parisinos, Chetan Parmar, Panna Patel, Samir Pathak, Stephen Pereira, Stephanos Pericleous, Rosemary Phillips, Tom Pike, Lushen Pillay, Joao Pinheiro, Parisa Pirjamali, Yanish Poolovadoo, Mariuca Popa, Sarah Powell-Brett, Melissa Prior-Ong, David Propper, Leonard Quinn, Khaled Radwan, Alyssa Ralph, Veena Ramachandran, Ganeshan Ramsamy, Hind Rassam, Anjana Ray-Chaudhuri, Srikanth Reddy, Shahriar Reza, Karim Rezk, Paul Rice, Lysia Richmond, Brianda Ripoll, Syed Rizvi, Sarah Robinson, Natalie Robson, Polly Rogers, Megan Rowley, Thomas Russell, Dana Safarova, Harkiran Sagoo, Maurice Samake, Sharukh Sami, Kumar Samraj, Panchali Sarmah, Edward Saxton, Bethany Scutt, Chaminda Sellahewa, Gourab Sen, Zara Shaida, Amrita Shandakumar, Nicholas Sharer, Syed Shaukat, Roosey Sheth, Guy Shingler, Amy Shroll, Ajith K. Siriwardena, James Skipworth, Sarah Slater, Conor Smith, Andrew Smith, Opeyemi Sogaolu, Claire Stevens, Duncan Stewart, Weronika Stupalkowska, Vikas Sud, Zain Sultan, Luke Summers, Nikhil Suresh, Jonathan Sutton, Wei Jian Tan, Chew Tan, Andrei Tanase, Andrei Tanase, Lulu Tanno, Luke Taylor, Mark Taylor, Rohan Thakkar, Donna Thomas, Emily Thompson, Benjamin Tinsley, Elizabeth Toy, David Tsang, Archil Tsirekidze, Dimitrios Tsironis, Sophie Tucker, Tracey Turner, Varu Udayachandran, Stijn van Laarhoven, Lakshmi Deepa Vandadi, Rebecca Varley, Darmarajah Veeramootoo, Suresh Vasan Venkatachalapathy, Ashwin Verma, Mark Vipond, Daniel Waite, Amy Ward, Ben Warner, Justin Waters, Alexander West, Douglas Whitelaw, Matthew Williams, Rhys Williams, Phoebe Wilson, Danylo Yershov, Alistair Young, Muneeb Zafar, Osama Zaman, Melissa Zhao

**Affiliations:** 1 College of Medical and Dental Sciences, University of Birmingham, Birmingham, England; 2 Queen Elizabeth Hospital, Birmingham, England; 3 Department of Academic Surgery, University of Birmingham, Birmingham, England; 4 Southend University Hospital, Southend-on-Sea, England; 5 Birmingham Surgical Trials Consortium, University of Birmingham, Birmingham, England; 6 The Christie NHS Foundation Trust, Manchester, England; 7 Cambridge University Hospitals NHS Foundation Trust, Cambridge, England; 8 Birmingham Centre for Observational and Prospective Studies, University of Birmingham, Birmingham, England; 9 St George’s Hospital, London, England; 10 Forth Valley Royal Hospital, Larbert, Scotland; 11 Luton and Dunstable University Hospital, Luton, England; 12 Craigavon Area Hospital, Craigavon, Northern Ireland; 13 Queen Alexandra Hospital, Portsmouth, England; 14 University Hospitals of Leicester NHS Trust, Leicester, England; 15 Freeman Hospital, Newcastle, England; 16 University Hospital of Wales, Cardiff, Wales; 17 Queen’s Hospital, Romford, England; 18 Whittington Hospital NHS Trust, London, England; 19 Bristol Royal Infirmary, Bristol, England; 20 Walsall Manor Hospital, Walsall, England; 21 Eastbourne District General Hospital, Eastbourne, England; 22 Darent Valley Hospital, Dartford, England; 23 Royal Lancaster Infirmary, Lancaster, England; 24 Hull University Teaching Hospitals NHS Trust, Hull, England; 25 Wexham Park, Frimley Health NHS Foundation Trust, Frimley, England; 26 Furness General Hospital, Barrow-in-Furness, England; 27 NHS Grampian, Aberdeen, Scotland; 28 Colchester General Hospital, Colchester, England; 29 Morriston Hospital, Swansea, Wales; 30 Airedale General Hospital, West Yorkshire, England; 31 Ipswich Hospital, Ipswich, England; 32 Inverclyde Royal Hospital, Greenock, Scotland; 33 The Royal Wolverhampton NHS Trust, Wolverhampton, England; 34 Derriford Hospital, Plymouth, England; 35 University Plymouth NHS Trust, Plymouth, England; 36 Southampton General Hospital, Southampton, England; 37 Royal London Hospital, London, England; 38 East Kent Hospitals University NHS Foundation Trust, Kent, England; 39 University Hospital North Midlands NHS Trust, Stoke-on-Trent, England; 40 Hinchingbrooke Hospital, Huntingdon, England; 41 Northampton General Hospital, Northampton, England; 42 Royal Derby Hospital, Derby, England; 43 North Tees and Hartlepool NHS Trust, Durham, England; 44 West Suffolk Hospital, Bury St Edmunds, England; 45 Bedfordshire Hospitals NHS Foundation Trust, Bedford, England; 46 Royal Gwent Hospital, Newport, Wales; 47 Altnagelvin Area Hospital, Derry, Northern Ireland; 48 Basingstoke & North Hampshire Hospital, Basingstoke, England; 49 Cumberland Infirmary, Carlisle, England; 50 Churchill Hospital, Oxford, England; 51 West Middlesex Hospital, Isleworth, England; 52 Broomfield Hospital, Broomfield, England; 53 Homerton Hospital, London, England; 54 James Paget Hospital, Great Yarmouth, England; 55 Victoria Hospital, Kirkcaldy, Scotland; 56 Frimley Park, Frimley Health NHS Foundation Trust, Frimley, England; 57 Royal Bournemouth Hospital, Bournemouth, England; 58 Manchester Royal Infirmary, Manchester, England; 59 Antrim Area Hospital, Antrim, Northern Ireland UK; 60 Gloucestershire Royal Hospital, Gloucester, England; 61 Conquest Hospital, East Sussex Healthcare Trust, Hastings, England; 62 Dumfries and Galloway Royal Infirmary, Cargenbridge, Scotland; 63 Royal Sussex County Hospital, Brighton, England; 64 Worcestershire Royal Hospital, Worcester, England; 65 Lister Hospital, Stevenage, England; 66 Hereford County Hospital, Hereford, England; 67 Royal Free Hospital, London, England; 68 Norfolk and Norwich University Hospital, Norwich, England; 69 Hammersmith Hospital, London, England; 70 ysbyty gwynedd, Bangor, Wales; 71 Glasgow Royal Infirmary, Glasgow, Scotland; 72 Chelsea & Westminster NHS Trust, London, England; 73 Royal Glamorgan Hospital, Ynysmaerdy, Wales; 74 Nottingham City Hospital, Nottingham, England; 75 Queen’s Hospital, Burton, England; 76 Royal Victoria Hospital, Belfast, Northern Ireland; 77 Kettering General Hospital, Kettering, England; 78 Royal Blackburn Hospital, Blackburn, England; 79 Torbay and South Devon NHS Foundation Trust, Torquay, England; 80 University Hospital of North Durham, Durham, England; 81 North Middlesex University Hospital NHS Trust, London, England; 82 St James’s University Hospital, Leeds, England; 83 Poole Hospital, Poole, England; 84 Sandwell General Hospital, Birmingham, England; 85 Wrexham Maelor, Wrexham, Wales; 86 Royal Liverpool University Hospital, Liverpool, England; 87 University College London Hospitals, London, England; 88 Huddersfield Royal Infirmary, Huddersfield, England; 89 Warrington and Halton Teaching Hospitals NHS Foundation Trust, Warrington, England; 90 Russells Hall Hospital, Birmingham, England; 91 Royal Devon and Exeter Hospital, Exeter, England; 92 Peterborough City Hospital, Peterborough, England; 93 Princess Alexandra Hospital, Redditch, England; 94 Maidstone and Tunbridge Wells NHS Trust, Royal Tunbridge Wells, England; 95 Northumbria Healthcare Trust, Northumbria, England

**Keywords:** Pancreatic cancer, Pancreatic cancer

## Abstract

**Introduction:**

CONTACT is a national multidisciplinary study assessing the impact of the COVID-19 pandemic upon diagnostic and treatment pathways among patients with pancreatic ductal adenocarcinoma (PDAC).

**Methods:**

The treatment of consecutive patients with newly diagnosed PDAC from a pre-COVID-19 pandemic cohort *(07/01/2019-03/03/2019)* were compared to a cohort diagnosed during the first wave of the UK pandemic (‘*COVID’ cohort, 16/03/2020-10/05/2020)*, with 12-month follow-up.

**Results:**

Among 984 patients (pre-COVID: *n* = 483, COVID: *n* = 501), the COVID cohort was less likely to receive staging investigations other than CT scanning (29.5% vs. 37.2%, *p* = 0.010). Among patients treated with curative intent, there was a reduction in the proportion of patients recommended surgery (54.5% vs. 76.6%, *p* = 0.001) and increase in the proportion recommended upfront chemotherapy (45.5% vs. 23.4%, *p* = 0.002). Among patients on a non-curative pathway, fewer patients were recommended (47.4% vs. 57.3%, *p* = 0.004) or received palliative anti-cancer therapy (20.5% vs. 26.5%, *p* = 0.045). Ultimately, fewer patients in the COVID cohort underwent surgical resection (6.4% vs. 9.3%, *p* = 0.036), whilst more patients received no anti-cancer treatment (69.3% vs. 59.2% *p* = 0.009). Despite these differences, there was no difference in median overall survival between the COVID and pre-COVID cohorts, (3.5 (IQR 2.8–4.1) vs. 4.4 (IQR 3.6–5.2) months, *p* = 0.093).

**Conclusion:**

Pathways for patients with PDAC were significantly disrupted during the first wave of the COVID-19 pandemic, with fewer patients receiving standard treatments. However, no significant impact on survival was discerned.

## Introduction

The COVID-19 pandemic has had an unprecedented impact on healthcare systems, with major impact upon delivery of non-COVID-related services [1]. Pressure on healthcare services to prioritise care for those with COVID-19 infection inevitably led to a reduction in service availability for patients with other conditions [2]: an estimated 28 million operations were cancelled worldwide in the first 12 weeks of the pandemic, for example [3].

Cancer patients were considered particularly vulnerable to COVID-19, due to increased risk of infection and mortality [4–7]. Initial data suggested that infection with COVID-19 in the perioperative period, or when receiving anti-cancer drug treatment, was associated with high rates of mortality [8, 9]. Consequently, at the start of the UK COVID-19 pandemic, guidelines were generated by both national and international groups regarding changes to standard cancer patient management [10, 11]. Specifically for patients with PDAC, the European Society for Medical Oncology (ESMO) [12] and UK Consensus Statement for treatment of pancreatic cancer [9] guidance made recommendations on modifying patient pathways, generally anticipating less or deferred surgery, a more cautious approach to the use of systemic therapy particularly in the case of unresectable disease, and an opportunity to explore hitherto non-standard hypofractionated radiotherapy regimens. Subsequent data did not confirm anticancer drug treatment to be associated with increased mortality, hence oncologists revised the initial plans to de-escalate use of these therapies in the second half of 2020 and subsequent waves of the pandemic [13].

PDAC is associated with some of the worst outcomes from any form of cancer [14, 15]. The benefits of anti-cancer interventions are modest relative to those achieved for most other common cancers, and in an unprecendented situation when healthcare resources needed prioritising towards those most likely to benefit (both with regard to COVID-19 infection and to cancer), there was a risk that patients with PDAC might have been particularly vulnerable to changes in standard of care that might in fact worsen their disease outcomes. The aim of the CONTACT study was to compare the recommended and received treatments among patients diagnosed with PDAC during the first wave of the COVID-19 pandemic with a similar patient cohort diagnosed in early 2019, pre-pandemic. The primary aim was to determine whether diagnosis of PDAC during the pandemic was associated with a reduction in standard treatment of PDAC, secondary aims were to compare treatment intent to received treatment and survival at one year.

## Methods and analysis

The CONTACT study is reported according to Strengthen the Reporting of Observational Studies in Epidemiology (STROBE) guidelines [16].

The primary objective was to compare treatment(s) received by patients with PDAC diagnosed during the first wave of the COVID-19 pandemic in the UK, compared with a similar cohort diagnosed prior to the pandemic. Secondary objectives include assessment of the diagnostic pathway, recommended treatment, times to treatment and 12-month outcomes, compared to a pre-pandemic cohort.

### Setting and study design

This was a national, observational cohort study that implemented a collaborative research model with data collection undertaken by trainee doctors. A novel, mixed prospective and retrospective design, with retrospective case identification of both cohorts was used. The pre-COVID cohort comprised patients diagnosed with PDAC during an 8 week period, between 07 January to 03 March 2019. The COVID cohort comprised patients diagnosed with PDAC during an 8 week period, between 16 March to 10 May 2020. All patients were followed up for 12 months, so the data collection on the pre-pandemic cohort predated the start of the UK COVID-19 pandemic.

All UK hospitals (*n* = 156) with an established PDAC multidisciplinary team (MDT) were eligible to join the study and were invited through email invitation and by invitation through specialty organisations (Pancreatic Society of Great Britain and Ireland, Association of Upper Gastrointestinal Surgery, and Great Britain and Ireland Hepatopancreatobiliary Association). PDAC treatment in the UK is via a ‘hub-and-spoke’ network whereby each specialist surgical ‘hub’ is networked to its ‘spoked’ hospitals that do not provide surgery. The definition of ‘specialist’ centre henceforth, refers to a hospital in which pancreatic surgery is available. Across most networks chemotherapy is delivered at the local ‘spokes’, although in two centres, the delivery of chemotherapy has been largely centralised. Volunteer trainee regional leads were recruited to oversee data collection at hospitals linked to their network. Medical students worked with the study coordinators to support the regional leads, facilitate communication and ensure data was collected according to the study protocol.

All adult patients (≥18 years old) with suspected PDAC presenting during the case identification periods and discussed at pancreatic cancer MDTs were included in this study. In the two regional sites (Manchester and Liverpool) with both centralised surgery and chemotherapy services, all patients were identified at the regional site. For all other sites, data was entered by the site where the patient was both initially diagnosed with PDAC and subsequently received ongoing treatment, such as chemotherapy given at the local site, ensuring treatment throughout the patient pathway as well as follow-up data was captured.

### Inclusion and exclusion criteria

Before analysis, the data was screened to ensure all included patients fulfilled the inclusion criteria for the study. Patients were included if they were over the age of 18 years, they had presented initially to the reporting hospital with suspected PDAC and had had an initial CT scan, indicative of such. Patients were excluded if subsequent investigations or treatment confirmed the diagnosis was not PDAC, or the data available was incomplete. A minimum data requirement of: receipt of index CT scan, MDT recommendation and treatment received, was used.

### Variables and data collection

The following data was collected: (1) baseline demographics, (2) diagnostic and staging tests, (3) management (both recommended treatment at the MDT and actual treatment received), and (4) survival at 12 months. Data was collected from routine medical records and no patients were contacted. Anonymised patient data was uploaded to a REDCap database [17, 18].

### Statistical methods

Descriptive statistics were used to display demographic variables. Continuous data was expressed as median (interquartile range; IQR), and categorical variables presented as numbers and/or percentages. Chi-squared test was used to test for significance in categorical variables whilst Mann–Whitney-*U* were used for ordinal and continuous data. Binary regression analysis was used to calculate odds ratios and corresponding 95% confidence intervals. Cox-regression analysis was used for hazard ratio calculation for 12-month survival, and a Kaplan–Meier logistic regression curve used to display the results. Due to data protection limitations, only the week of death was able to be collected. To mitigate bias, when calculating survival only, the date of the initial CT scan was adjusted to the Monday of that week, and ‘day of death’ assigned to the Monday of the week of death collected. A *p*-value of <0.05 was considered significant.

### Ethics and dissemination

Patient consent was not required for this study, as only routinely collected datapoints were collected by members of the local healthcare team, and the centrally analysed data was anonymous. This was confirmed using the national UK decision-making tool of the NHS Health Research Authority and the Medical Research Council [19]. The CONTACT study was locally registered as a clinical audit or service evaluation project at each participating site prior to patient identification and data collection.

## Results

### Baseline demographics

After screening 1261 possible cases and applying exclusion criteria, 984 cases with PDAC treated across 96 hospitals were included in the final analysis (pre-COVID: *n* = 484 and COVID: *n* = 501). 22 hospitals were specialist pancreatic centres (184 patients vs. 200 patients), and two networks centralised delivery of anti-cancer therapy (31 patients vs. 31 patients). There were no significant differences in median age (73 years, range: 65–80 vs. 73 years, range: 66–81), gender (268, 53.4% vs. 258, 53.4% male), or performance status (PS: 320, 63.8% vs. 290, 59.9% PS 0–1) between the COVID and pre-COVID cohorts. The vast majority of patients were considered to be on a non-curative pathway (424, 84.6% of COVID vs. 389, 80.5% of pre-COVID cohort). A complete list of baseline characteristics is shown in Table 1.

**Table 1:** Baseline demographics.

		Pre-COVID *n* = 486	COVID *n* = 501	*p*-value
Median age, years (IQR)		73 (66–81)	73 (65–80)	0.665
Age quintile, years	<60	70 (14.4%)	71 (14.1%)	0.705
	60–70	122 (25.2%)	134 (26.7%)	
	71–75	88 (18.2%)	83 (16.5%)	
	76–80	77 (15.9%)	96 (19.1%)	
	>80	126 (26%)	117 (23.3%)	
Gender	Male	258 (53.4%)	268 (53.4%)	0.981
WHO/ECOG performance status	0	153 (31.6%)	165 (32.9%)	0.521^a^
	1	137 (28.3%)	155 (30.9%)	
	2	101 (20.9%)	81 (16.1%)	
	>3	92 (19%)	100 (19.9%)	
Country	England	381 (78.8%)	393 (78.4%)	0.241
	Scotland	49 (10.1%)	38 (7.5%)	
	Wales	22 (4.5%)	34 (6.7%)	
	Northern Ireland	31 (6.4%)	36 (7.1%)	
Index multiple deprivation quintile^b^	1	94 (20.6%)	95 (20%)	0.704^a^
	2	82 (17.9%)	79 (16.6%)	
	3	111 (24.3%)	132 (27.7%)	
	4	100 (21.9%)	84 (17.6%)	
	5	69 (15.1%)	85 (17.8%)	
Body mass index^c^	Underweight	32 (7.1%)	44 (9.5%)	0.152^a^
	Normal	263 (59.1%)	275 (59.7%)	
	Overweight	98 (22%)	96 (20.8%)	
	Moderately obese	38 (8.5%)	39 (8.4%)	
	Severely obese	9 (2%)	5 (1%)	
	Very severely obese	5 (1.1%)	1 (0.2%)	
Charlson comorbidity index score	<5	353 (73.08%)	355 (70.85%)	0.429^a^
	5–7	114 (23.6%)	127 (25.34%)	
	>7	16 (3.31%)	19 (3.79%)	
Jaundiced	*N* (%)	186 (39%)	195 (39.7%)	0.839
Pathway intent				
Curative	*N* (%)	94 (19.5%)	77 (15.4%)	0.09
Non-curative	*N* (%)	389 (80.5%)	424 (84.6%)	

Data are reported as median (IQR), with *p*-value from Mann–Whitney *U*-test, or as *N* (%), with *p*-value from chi-square test, unless stated otherwise.

^a^Ordinal Data *p*-values from Mann–Whitney-*U*-test.

^b^*n* = 456, 94.4% pre-COVID and *n* = 475, 94.8% COVID.

^c^*n* = 445, 92.2% pre-COVID and *n* = 460, 91.8% COVID.

### Staging investigations and treatment of jaundice

All patients underwent at least one CT scan as per the inclusion criteria. Patients in the COVID cohort were less likely to undergo any further staging tests compared with pre-COVID (148/501, 29.5% vs. 180/486, 37.2%; *p* = 0.01). Specifically, there was a reduction in the use of EUS (OR: 0.65, 95%CI: 0.48–0.88; *p* = 0.006) and MRI (OR: 0.66, 95%CI: 0.44–0.99; *p* = 0.043) compared to those patients in the pre-COVID cohort, and fewer patients had a histologically confirmed diagnosis of malignancy (OR: 0.72, 95%CI: 0.56–0.93, *p* = 0.011). There was no difference in the time from diagnosis to the various staging investigations between the cohorts. There was no difference in the proportion of jaundiced patients, treatment of jaundice or time to treating jaundice between the cohorts (Table 2).

**Table 2:** Staging and investigations and treatment of jaundice.

		Pre-COVID *n* = 486	COVID *n* = 501	*p*-value
Any staging test other than CT	*N* (%)	180 (37.2%)	148 (29.5%)	**0.01**
EUS	*N* (%)	125 (25.8%)	93 (18.5%)	**0.006**
	Median time to Ix (IQR) (*n* = 191)	21.5 (12–33.75)	20 (10–36)	0.908
MRI	*N* (%)	64 (13.2%)	46 (9.1%)	**0.043**
	Median time to Ix (IQR) (*n* = 105)	9 (2–19)	7 (1.75–29.5)	0.346
PET-CT	*N* (%)	49 (10.1%)	38 (7.5%)	0.368
	Median time to Ix (IQR) (*n* = 82)	29 (12–42)	26 (17–41)	0.665
Pathological diagnosis of malignancy^a^	*N* (%)	256 (53%)	225 (44.9%)	**0.011**
Biliary drainage^b^	Percutaneous	32 (6.7%)	28 (5.7%)	0.497
	Endoscopic	140 (29.4%)	160 (32.5%)	0.315
	No drainage	14 (2.9%)	7 (1.4%)	0.103
	Median time to Drainage (IQR) (*n* = 337)	9 (4–20)	7 (3–16)	0.106

Data are reported as median (IQR), with *p*-value from Mann–Whitney *U*-test, or as *N* (%), with *p*-value from chi-square test, unless stated otherwise.

^a^Pathological diagnosis refers to either cytological/histological or both, by way of fine needle aspiration, tissue biopsy or biliary cytology.

^b^*n* = 476, 97.9% pre-COVID and 88 *n* = 491, 98.0% COVID.

Bold *p*-values are those which have a significant value of <0.05.

### MDT-recommended treatment of PDAC

Across the whole study population (pre-COVID *n* = 483; COVID *n* = 501) more patients were recommended best supportive care (i.e., no surgery or non-surgical anticancer treatment) in the COVID cohort (44.5% vs. 34.4% *p* = 0.001; OR 1.53 95%CI 1.18–1.98), fewer were recommended cancer resection surgery (8.4% vs. 14.9% *p* = 0.002; OR 0.52 95%CI 0.35–0.78), while more were recommended upfront chemotherapy (UFC) (7.0% vs. 4.6% *p* = 0.105; OR 1.57, 95%CI 0.91–2.7) in the COVID cohort compared to the pre-COVID cohort. There was also a non-significant reduction in the recommendation to use palliative chemotherapy (40.1% vs. 46.2% p = 0.056; OR 0.78, 95%CI 0.61–1.01) in the COVID cohort.

### Actual treatment of PDAC

**Fig. 1:**
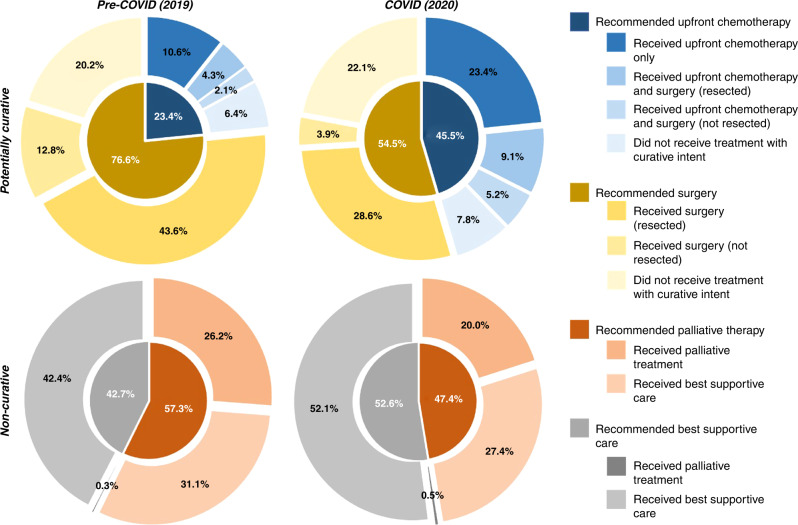
Multilevel pie-charts to illustrate the change in proportions of patients following a potentially curative (top), or non-curative (bottom) and subsequent treatments, from pre-COVID (left), to during the pandemic (right). The inner circles represent the proportions of patients recommended to respective management pathways, whilst the outer circles represent the proportions of subsequent treatment, or lack thereof, received.

Across the whole study population, more patients received best supportive care (67.3% vs. 59.2% *p* = 0.009; OR 1.42, 95%CI: 1.09–1.84), fewer underwent cancer resection surgery (6.4% vs. 9.3% *p* = 0.036; OR 0.64, 95%CI: 0.37–0.97) and more received UFC (5.8% vs. 3.3% *p* = 0.063; OR 1.81) in the COVID cohort compared with the pre-COVID cohort. There was also a non-significant trend in reduction in the use of palliative chemotherapy (17.4% vs. 21.3% *p* = 0.116; OR 0.76, 95%CI: 0.56–1.07) in the COVID cohort. The intended and actual treatments received for the whole cohort are summarised in Fig. 1.

### Survival analysis

**Fig. 2:**
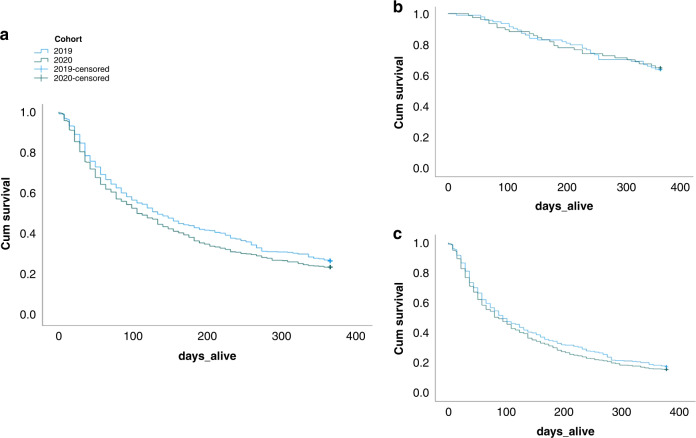
Kaplan–Meier Curves to show overall survival (in days) at 12-month follow up, for Pre-COVID (2019; blue) vs  COVID (2020; green). X axis shows number of days, whilst Y shows cumulative survival. **a** Survival curve of the whole study cohort, **b** Survival curve of patients on the potentially curative pathway and **c** survival curve of patients on the non-curative pathway.

There was no difference in survival between the two cohorts overall, with a median survival of 3.5 (IQR 2.8–4.1) months in the COVID cohort vs. 4.4 (IQR 3.6–5.2) months in the pre-COVID cohort (HR 1.132, 95%CI 0.980–1.037; *p* = 0.093) (Fig. 2); 23.4% and 26.5% of the COVID and pre-COVID cohorts were alive at 12 months. Comparing COVID and pre-COVID cohorts, 64.9% and 63.8% of patients within the potentially curative pathway were alive at 12-months (HR: 0.98, 95%CI: 0.59–1.62; *p* = 0.932) whilst 9.9% and 11.3% of patients within the non-curative pathway were alive at 12-months (HR: 1.055, 95%CI: 0.85–1.31; *p* = 0.624).

### Within group treatment

Patients could follow either a pathway with curative or non-curative intent and these are now considered separately.

### Curative intent pathway

Fewer patients on a curative pathway in the COVID cohort vs. pre-COVID cohort were recommended surgery (42/77, 54.5% vs. 72/94, 76.6% *p* = 0.001) whilst more were recommended UFC (35/77, 45.5% vs. 22/94, 23.4% *p* = 0.002). However, in the groups recommended surgery there was no difference in the proportion that were resected (22/42, 52.3% vs. 41/72, 56.9%: COVID vs. pre-COVID, respectively; *p* = 0.636). Thus, 47.7% patients in the COVID cohort and 43.1% patients in the pre-COVID cohort recommended surgery were not actually operated on.

Patients with good PS (0–1) were more likely to be recommended to receive UFC during the pandemic, rather than immediate surgery (46.5% vs. 20.7%, *p* < 0.001). The number of patients recommended UFC with resectable tumours (rather than borderline or locally advanced) also increased during the pandemic, though non-significantly (24.2% vs. 5.9%, *p* = 0.109).

There was no difference in the proportion of jaundiced patients or those proceeding direct to surgery with jaundice between the cohorts. There was no difference in the use of, or time to, adjuvant therapy, but patients were more likely to receive single agent chemotherapy in the COVID cohort (6/16, 38% vs. 3/29, 8%, *p* = 0.038). Of those recommended UFC, only 20% in the COVID (*n* = 7/35) and 18% in the pre-COVID (*n* = 4/22) cohorts were resected. A further 11% and 9% in the COVID (*n* = 4/35) and pre-COVID (*n* = 2/22) cohorts underwent attempted surgery but failed resection after UFC (Table 3). There was no difference in vascular resection rate, T or N status between the operated cohorts. There was no difference in the rates of local or metastatic recurrence, of the time to recurrence between the operated cohorts, nor 90-day-mortality after resection surgery (2/29, 6.9% vs. 1/45, 2.2%, *p* = 0.557).

**Table 3:** Patients on a pathway of curative intent.

		Pre-COVID *n* = 94	COVID *n* = 77	*p*-value
Whole cohort *n* = 171	*Jaundiced*	62 (65.9%)	54 (70.1%)	0.561
	*Recommended Surgery*	72 (76.6%)	42 (54.5%)	**0.001**
	*Recommended Neoadjuvant Therapy*	22 (23.4%)	35 (45.5%)	**0.002**
Cohort recommended		*n* = 72	*n* = 42	
surgery only *n* = 114	*Direct to surgery with jaundice*	11 (15.3%)	4 (9.5%)	0.411
	*Did not undergo any surgery*	19 (26.4%)	17 (40.5%)	0.177
	*Resected*	41 (56.9%)	22 (52.4%)	
	*Not Resected at surgery*	12 (16.7%)	3 (7.1%)	
	*Median time to Surgery days (IQR)*	45 (19.8–74.8)	59 (36.5–95.0)	0.106
Upfront Resected cohort only^a^ *n* = 63		*n* = 41	*n* = 22	
	*Adjuvant Chemotherapy*	29 (72.5%)	16 (72.7%)	0.656
	*FOLFIRINOX*	11 (26.8%)	5 (22.7%)	**0.038**
	*Gem* *+* *Cap*	15 (36.6%)	5 (22.7%)	
	*Gemcitabine or capecitabine*	3 (7.3%)	6 (27.3%)	
	*Completed full allocation of cycles*	22 (55.0%)	12 (54.5%)	0.949
	*Median Time surgery-adjuvant therapy (weeks, IQR)*	10 (8.8–12.6)	10.1 (7.1–12.1)	0.461
Cohort recommended UFC only *n* = 57		*n* = 22	*n* = 35	
UFC status	*Borderline/Locally Advanced*	20 (90.9%)	26 (74.3%)	0.122
	*Resectable*	2 (9.1%)	9 (25.7%)	
UFC therapy received	*Chemotherapy Alone*	11 (50%)	24 (68.6%)	0.481
	*Chemoradiotherapy*	4 (18.2%)	3 (8.6%)	
	*Radiotherapy Alone*	1 (4.5%)	2 (5.7%)	
	*No NAT received*	6 (27.3%)	6 (17.1%)	
Progression to surgery	*Did not undergo any surgery*	16 (72.3%)	24 (68.6%)	0.901
	*Resected*	4 (18.1%)	7 (20%)	
	*Not Resected at surgery*	2 (9%)	4 (11.4%)	

Data are reported as median (IQR), with *p*-value from Mann–Whitney *U-*test, or as *N* (%), with *p*-value from chi-square test;

^a^These cohorts do not include patients who underwent surgery after upfront chemotherapy (UFC).

Bold *p*-values are those which have a significant value of <0.05.

### Non-curative intent pathway

Fewer patients on a non-curative pathway in the COVID cohort were recommended palliative therapy (COVID 201/424, 47.4% vs. pre-COVID 223/389, 57.3% OR: 1.11 95%CI: 0.96–1.85; *p* = 0.001), fewer patients actually received *any* palliative therapy (20.5% vs. 26.5%; OR:0.72 95%CI: 0.52–0.99, *p* = 0.045), and fewer received palliative chemotherapy (18.9% vs. 24.7%; OR: 0.71 95%CI: 0.51–0.99, *p* = 0.044). In almost half of cases in both cohorts (48.6% COVID vs. 45.4% pre-COVID), the reason given for patients not receiving recommended palliative therapy was frailty. The next most common reasons were recurrence/progression (26.4% COVID vs. 29.0% pre-COVID) and patient choice (18.6% COVID vs. 20.2% pre-COVID). COVID-19 itself was only cited as a reason in 2.3% of the COVID cohort.

There was no difference in rates of palliative radiotherapy between the COVID and pre-COVID cohorts (3.5% vs. 3.3%; *p* = 0.878), or reasons provided why patients were deemed non-curative/unresectable, *p* = 0.363. Among patients receiving palliative chemotherapy, there was no difference in the first-line agents received, the proportion of patients completing all cycles of chemotherapy, median time to starting chemotherapy, or the proportion of patients receiving second line therapy. This data is described in detail in Table 4. Patients with poor performance status (≥2) were less likely to be recommended to receive palliative therapy during the pandemic (25.1% vs. 38.1%, *p* = 0.009). There was no difference for the reasons given why patients did not receive any therapy between the cohorts (COVID *n* = 337/424, 79.5%, pre-COVID *n* = 286/389, 73.5%; *p* = 0.277).

**Table 4:** Patients on a pathway of non-curative intent.

		Pre-COVID *n* = 389	COVID *n* = 424	*p*-value
Whole cohort *n* = 813	Recommended Palliative Therapy	223 (57.3%)	201 (47.4%)	**0.004**
	Received palliative therapy	103 (26.5%)	87 (20.5%)	**0.045**
	Recommended best supportive care	166 (42.7%)	223 (52.6%)	
	Received best supportive care	286 (73.5%)	337 (79.5%)	
	Received palliative chemotherapy	96 (24.7%)	80 (18.9%)	**0.044**
	Received palliative radiotherapy	13 (3.3%)	15 (3.5%)	0.878
	Palliative surgery	4 (1.7%)	1 (0.4%)	0.217
Reason for non-curative pathway selection	Metastatic disease	254 (65.2%)	251 (59.1%)	0.363
	Locally advanced disease	76 (19.5%)	88 (20.7%)	
	Potentially resectable but not offered surgery due to:			
	-Performance status	39 (10.0%)	55 (12.9%)	
	-Patient choice	8 (2.0%)	14 (3.3%)	
	-Unknown	12 (3.0%)	16 (3.7%)	
Cohort that received palliative chemotherapy only *n* = 176		*n* = 96	*n* = 80	
	FOLFIRINOX	29 (30.2%)	38 (47.5%)	0.088
	Gem/Cap	15 (15.6%)	13 (16.3%)	
	Gem/Abraxane	21 (21.9%)	10 (12.5%)	
	Gemcitabine or capecitabine	27 (28.1%)	14 (17.5%)	
	Other	4 (4.2%)	5 (6.3%)	
	Completed full allocation of cycles	37 (38.5%)	33 (41.3%)	0.625
	Median time to chemotherapy days (IQR)	52 (42.3–73.3)	49 (41.3–75.5)	0.699
	2nd line palliative chemotherapy received	12 (12.5%)	10 (12.5%)	0.954
Cohort that received no palliative therapy, after recommendation. *n* = 623		*n* = 286	*n* = 337	
	Frailty	130 (45.4%)	164 (48.6%)	0.277
	Patient choice	58 (20.2%)	63 (18.6%)	
	Recurrence/progression	83 (29%)	89 (26.4%)	
	COVID	0 (0%)	8 (2.3%)	
	Unknown	15 (5.2%)	13 (3.8%)	

Data are reported as median (IQR), with *p*-value from Mann–Whitney *U-*test, or as *N* (%), with *p*-value from chi-square test.

Bold *p*-values are those which have a significant value of <0.05.

## Discussion

The CONTACT study was an observational study of two cohorts of consecutive patients diagnosed with PDAC across UK hospitals with 12 months follow-up, before and during the first wave of the COVID-19 pandemic. Importantly, there was no overlap of the follow-up period for the 2019 cohort and the start of the pandemic. Both cohorts shared similar demographics typical of this disease, being relatively elderly (40% over the age of 75 years, 25% over the age of 80 years) and frail (35–40% PS 2–3) populations, and only 1 in 5 being considered potentially curable at the time of initial assessment.

The key CONTACT study findings confirmed that access to surgery during the pandemic was significantly curtailed and patients were offered up-front chemotherapy as a bridging treatment modality, aimed at deferring planned surgery, as recommended in the UK consensus recommendations [9]. In addition, the proportion of patients on a non-curative pathway who received palliative therapy was lower compared with the pre-COVID cohort. Despite these differences, survival at 12-months for the two cohorts overall did not differ significantly. Other notable observations are discussed in three sections: diagnosis, curative intent pathway and non-curative intent pathway.

The National Institute of Health and Care Excellence (NICE) guidance recommends resectional surgery rather than pre-operative drainage among jaundiced patients with potentially resectable disease [11]. However, ESMO guidelines for management of PDAC during COVID-19 recommended prompt resolution of jaundice to create better conditions for subsequent management, be that curative or palliative [12]. The proportion of jaundiced patients proceeding direct to surgery was low, and did not differ between the cohorts. Although there was no significant change in the apparent treatment of jaundice, which requires invasive and possibly aerosol generating procedures, there was a reduction in the use of additional staging tests (EUS and MRI) among patients in the COVID cohort, raising concern that some patients may have been inadequately managed.

Among patients treated with curative intent there was an increase in the use of UFC during COVID. Whilst NICE guidance predating COVID-19 recommends neoadjuvant chemotherapy (NAT) to be used within a trial-based setting, emerging evidence has demonstrated potential benefits of NAT in patients with potentially resectable disease [20]. NAT may increase the number of patients completing their treatment course [21]. During the pandemic, with increased risk of perioperative morbidity, and reducing in access to surgery [22], UFC was by necessity used as a ‘bridging’ modality to delay surgery and hence became a real-time strategy to ‘test cancer biology’ [23], beyond its use with pure neoadjuvant intent. However, immunosuppressive risks of chemotherapy must also be considered, particularly in this vulnerable cohort, with an increased risk of mortality with COVID-19 infection, when compared to a non-cancer population [24]. Regardless of the cohort, the rates of resectional surgery following pre-operative chemotherapy were very low, with 20% or fewer patients being resected ultimately, either during or pre-COVID. These UK data compare poorly with the 61% resection rate reported in the Dutch PREOPANC phase III trial for patients with resectable or borderline resectable disease receiving neoadjuvant chemoradiotherapy [25]. The reasons for discrepancy in these figures are not entirely clear, but most likely reflect real-world data, lacking the rigors of a prospective controlled trial. UFC may well have been recommended for patients with more extensive, locally advanced disease, in whom resection rates are known be much lower.

The rapid uptake of UFC, as well as hypofractionated radiotherapy, not previously commissioned has demonstrated the potential value of real-world evaluation of new patient pathways that would historically have only be tested within prospective randomised trials. Clinical trials are expensive, time consuming and, as demonstrated by the ESPAC5F randomised trial of NAT, can be extremely challenging to recruit to [26]. It is noteworthy that since the pandemic, NAT is being adopted for patients with borderline resectable PDAC and outcome data for this patient group should be formally assessed.

For those patients completing surgery, whilst there was no difference in the proportion of patients receiveing adjuvant chemotherapy, there was a difference in the regimens used, with an increase in the use of single agent chemotherapy regimens in the COVID cohort. The NICE PDAC management guidelines predate the PRODIGE-24 trial demonstrating superiority of adjuvant mFOLFIRINOX compared with adjuvant gemcitabine, but do recommend combination chemotherapy (gemcitabine+capecitabine) in preference to gemcitabine, based on randomised trial evidence of benefit [11]. The decision to offer patients single agent rather than combination chemotherapy may well have been a deliberate decision to reduce risk of myelosuppression and safeguard patients. Reassuringly, the proportion of patients starting and completing adjuvant therapy was high and did not differ between the cohorts. The number of patients commencing adjuvant therapy in the present study, compares favourably to other national-scale data [21, 27–30]. Finally, there was no difference in time to initiation of adjuvant therapy, with median weeks to therapy in both cohorts being within 12-weeks of surgery, benefits of which are evidenced by data from published ESPAC trials [31, 32].

For patients on a non-curative pathway, there was a significant reduction in the use of palliative chemotherapy during the pandemic compared to pre-pandemic use. FOLFIRINOX was used first-line in the majority of cases in both cohorts, as per NICE guidance [11], and amongst patients who received palliative chemotherapy, there was a significant increase in FOLFIRINOX use (*p* = 0.044). Given that the number of patients recommended surgery was lower in the COVID cohort, whilst significantly more patients were recommended UFC, it is likely that more good performance status patients with locally advanced PDAC were being recommended and subsequently offered multi-agent chemotherapy. The lower use of palliative chemotherapy during the COVID-19 first wave may be explained by the perceived greater risk of harm from COVID-19 infection due to immunosuppression whilst on chemotherapy. A national consensus recommended a highly selective and individualised approach to palliative chemotherapy in the pandemic, with early response assessments encouraged to limit risk [9]. Interestingly the UK guidance was also to limit offering 2nd line chemotherapy, but in both patient cohorts the proportion of patients receiving 2nd line therapy was the same, at 12.5%. Similar rates of cycle completion before and during the pandemic may indicate persistent, careful selection of patients who would both benefit from, and tolerate palliative chemotherapy.

The proportion of patients receiving the intervention they were initially recommended at MDT was low both before and during the pandemic, particularly for those recommended to surgery (COVID 59.5% vs. pre-COVID 73.6%) and palliative therapy (COVID 43.3% vs. pre-COVID 46.2%). These data demonstrate limitations of MDT assessments, especially in the palliative setting, when key information regarding factors such as patient frailty and co-morbidities are generally lacking, but play an important part in determining fitness for intervention in an elderly population. Furthermore, the aggressive nature of PDAC can rapidly change patients’ overall health status and influence the intended treatment recommendations.

Whilst the pandemic forced changes upon PDAC patient treatment pathways, which otherwise would not have occurred over the past two years, some are not entirely detrimental; presenting opportunities to evaluate novel or emerging interventions. Some changes in managing patients have been serendipitous as they may not have occurred otherwise, such as a move to more remote consultations on an individualised basis. Exploring the role and outcomes of UFC in the pre-operative setting is another example; however, as seen in this study, the rates of resection were low and thus more work is needed to evaluate the role of UFC, specifically NAT, in managing early stages of PDAC. NHS England has drafted new guidance for faster diagnosis of PDAC. Soon to be published, implementation of this guidance will be essential to improving patient pathways and remove the delays that likely impact on patients accessing optimal care.

Given the changes to PDAC patient management during COVID vs. pre-COVID, it is surprising, but somewhat reassuring, that overall survival was not affected. It is possible that this may be due to a type 2 error, as only the minority of patients (~35%) received any form of treatment and the cohorts were relatively small, being identified over a 2 month period. On the other hand, the absolute benefits of both surgical and non-surgical interventions for this aggressive cancer type must be acknowledged to be modest, and these data give impetus for the urgent need to pursue research into its biology, understanding the drivers and develop innovative approaches to treatment as well as prevention.

Regional variation was not assessed in this study, but there was evidence of variation in practice between specialist and non-specialist sites and between regions in the RICOCHET national prospective PDAC audit [33, 34]. Further work will be required to assess whether different models of care were more or less successful at maintaining treatment throughout the pandemic with consequent impact on patient outcomes.

## Limitations and recommendations

It is a requirement to discuss all suspected and newly diagnosed cancer patients at MDT, therefore case ascertainment of PDAC patients is expected to be 100% at participating sites. Datapoints in this study were not validated, but it is known that when using the same network to capture data in the RICOCHET national PDAC audit that data accuracy exceeded 95% [33, 34]. Even so, the size of this study population is relatively modest. Not all centres with an MDT contributed data, so this is not a complete picture of care across the UK, but data is drawn from almost every specialist centre and from all the devolved nations, not just England. Given the relatively low rates of surgery among those affected by PDAC, the small size of some subgroups within this study limit interpretation. Access to national datasets might be expected to better quantify changes in pathways and treatments particularly on the key outcome of patient survival. The data are, however, strong enough to inform practice, with recommendations to: (1) ensure resources are available to adequately stage potentially resectable PDAC; (2) improve the quality of patient demographic information provided to MDTs in order to to make appropriate and accurate treatment pathway recommendations; (3) implement fast-track diagnosis and treatment pathways to minimise treatment delays; (4) prioritise PDAC patient access to palliative care support services both in hospitals and the community and (5) embrace research to improve outcomes of both early and advanced PDAC, given poor outcomes assosociated with this disease, irrespective of stage or interventions.

In summary, the CONTACT study reports a marked reduction in the staging and treatment provided to patients with PDAC diagnosed during the first wave of the UK COVID-19 pandemic. Though there was evidence of a change in pathway towards planned pre-operative chemotherapy prior to surgery among patients on a curative pathway during the pandemic, overall, this did not feed through to higher surgical resection rates. These data, essentially constituting a real-world experiment, strongly support the need now to prospectively assess the role of NAT and surgery in early PDAC. The overall findings that survival outcomes at 12 months were no different for patients whose standard pathways were modified compared with those whose pathways were not is a sobering signal that better treatments are urgently needed.

## Supplementary information

Supplementary File 1

Supplementary File 2

## Data Availability

The data that support the findings of this study are available from the corresponding author upon reasonable request.
