# Supplementary material for: The impact of the COVID-19 pandemic upon pancreatic cancer treatment (CONTACT Study): a UK national observational cohort study
Source: Br J Cancer. 2023 Mar 23;128(10):1922–32. doi: 10.1038/s41416-023-02220-2 (PMC10035482; doi:10.1038/s41416-023-02220-2)
Supplement: Supplementary file 1 [file 41416_2023_2220_MOESM1_ESM.docx]

| **Role** | **Institution** | **Title** | **First Name** | **Middle Initial (s)** | **Last Name** |
| --- | --- | --- | --- | --- | --- |
| Collaborator | Addenbrooke's Hospital | Mr | Asif |  | Jah |
| Collaborator | Addenbrooke's Hospital | Mr | Fanourios |  | Georgiades |
| Collaborator | Addenbrooke's Hospital | Mr | Michael |  | Feretis |
| Collaborator | Airedale General Hospital | Mr | Amro |  | Alamassi |
| Collaborator | Airedale General Hospital | Dr | Karim |  | Rezk |
| Collaborator | Airedale General Hospital | Mr | Madhu |  | Hebbar |
| Collaborator | Altnagelvin Area Hospital | Dr | John |  | McGoran |
| Collaborator | Altnagelvin Area Hospital | Dr | Richard |  | Howard |
| Collaborator | Altnagelvin Area Hospital | Dr | Neil |  | Bradley |
| Collaborator | Antrim Area Hospital | Dr | Leah |  | Gilroy |
| Collaborator | Antrim Area Hospital | Dr | Kerri-Marie |  | Heenan |
| Collaborator | Antrim Area Hospital | Dr | Gary |  | Morrison |
| Collaborator | Antrim Area Hospital | Dr | Hannah |  | McCaughan |
| Collaborator | Antrim Area Hospital | Dr | Rebecca |  | O'Kane |
| Collaborator | Antrim Area Hospital | Dr | Kok |  | Diong |
| Collaborator | Basingstoke & North Hampshire Hospital | Dr | Corinne |  | Brooks |
| Collaborator | Basingstoke & North Hampshire Hospital | Dr | Moeed |  | Minto |
| Collaborator | Basingstoke & North Hampshire Hospital | Mrs | Miriam |  | Jones |
| Collaborator | Bedfordshire Hospitals NHS Foundation Trust | Miss | Weronika |  | Stupalkowska |
| Collaborator | Bedfordshire Hospitals NHS Foundation Trust | Miss | Katharine |  | Bevan |
| Collaborator | Bedfordshire Hospitals NHS Foundation Trust | Dr | Zara |  | Shaida |
| Collaborator | Bedfordshire Hospitals NHS Foundation Trust | Dr | Sahra |  | Indayare |
| Collaborator | Bristol Royal Infirmary | Mr | James |  | Skipworth |
| Collaborator | Bristol Royal Infirmary | Mr | Reyad |  | Abbadi |
| Collaborator | Bristol Royal Infirmary | Mr | Samir |  | Pathak |
| Collaborator | Bristol Royal Infirmary | Dr | Stephen |  | Falk |
| Collaborator | Bristol Royal Infirmary | Miss | Caitlin |  | Jordan |
| Collaborator | Bristol Royal Infirmary | Mr | Stijn |  | van Laarhoven |
| Collaborator | Broomfield Hospital | Dr | Aarini |  | Mahalingam |
| Collaborator | Broomfield Hospital | Dr | Keval |  | Naik |
| Collaborator | Broomfield Hospital | Ms | Jennifer |  | Clark |
| Collaborator | Broomfield Hospital | Miss | Karen |  | Lloyd-Jones |
| Collaborator | Broomfield Hospital | Mrs | Irene |  | Charlesworth |
| Collaborator | Chelsea & Westminster NHS Trust | Dr | Neerav |  | Joshi |
| Collaborator | Chelsea & Westminster NHS Trust | Dr | Diya |  | Kapila |
| Collaborator | Chelsea & Westminster NHS Trust | Dr | Natasha |  | Kelly |
| Collaborator | Chelsea & Westminster NHS Trust | Miss | Clara |  | Hallinan-Rhodes |
| Collaborator | Chelsea & Westminster NHS Trust | Dr | Sujit |  | Mukherjee |
| Collaborator | Chelsea & Westminster NHS Trust | Dr | Roosey |  | Sheth |
| Regional Lead | The Christie NHS Foundation Trust | Dr | Rohan |  | Shotton |
| Collaborator | The Christie NHS Foundation Trust | Dr | Sarah |  | Kreppel |
| Collaborator | The Christie NHS Foundation Trust | Dr | Kathleen |  | Connors |
| Collaborator | The Christie NHS Foundation Trust | Dr | Philip |  | Hayton |
| Collaborator | Churchill Hospital Oxford | Dr | Ganeshan |  | Ramsamy |
| Collaborator | Churchill Hospital Oxford | Dr | Nikhil |  | Lal |
| Collaborator | Churchill Hospital Oxford | Dr | Patrick |  | Chen |
| Collaborator | Churchill Hospital Oxford | Mr | Carlo |  | Ceresa |
| Collaborator | Churchill Hospital Oxford | Mr | Srikanth |  | Reddy |
| Collaborator | Churchill Hospital Oxford | Mr | Vikas |  | Sud |
| Collaborator | Colchester General Hospital | Mr | Donald |  | Menzies |
| Collaborator | Colchester General Hospital | Miss | Tamsin |  | Morrison |
| Collaborator | Colchester General Hospital | Mr | Jonathan |  | Cormack |
| Collaborator | Colchester General Hospital | Dr | Anna |  | Palepa |
| Collaborator | Colchester General Hospital | Dr | Ouiam |  | Akotat |
| Collaborator | Conquest Hospital East Sussex Healthcare Trust | Dr | Steven |  | Fong |
| Collaborator | Conquest Hospital East Sussex Healthcare Trust | Dr | Victoria |  | Donovan |
| Collaborator | Conquest Hospital East Sussex Healthcare Trust | Dr | Yaser |  | Mohammed |
| Collaborator | Conquest Hospital East Sussex Healthcare Trust | Dr | Zain |  | Mitha |
| Collaborator | Conquest Hospital East Sussex Healthcare Trust | Dr | Dana |  | Safarova |
| Collaborator | Conquest Hospital East Sussex Healthcare Trust | Dr | Samuel |  | Ololade |
| Collaborator | Craigavon Area Hospital | Mr | Emmanouil |  | Epanomeritakis |
| Collaborator | Craigavon Area Hospital | Mrs | Maureen |  | Connolly |
| Collaborator | Craigavon Area Hospital | Dr | Paul |  | Rice |
| Regional Lead | Craigavon Area Hospital | Dr | Richard |  | Fox |
| Collaborator | Cumberland Infirmary (North Cumbria) | Dr | Badreldin |  | Mohamed |
| Collaborator | Cumberland Infirmary (North Cumbria) | Dr | Deepika |  | Natarajan |
| Collaborator | Cumberland Infirmary (North Cumbria) | Professor | Ruben |  | Canelo |
| Collaborator | Darent Valley Hospital | Dr | Ben |  | Warner |
| Collaborator | Darent Valley Hospital | Dr | Olaolu |  | Olabintan |
| Collaborator | Darent Valley Hospital | Dr | Yusria |  | Abukar |
| Collaborator | Darent Valley Hospital | Dr | Parisa |  | Pirjamali |
| Collaborator | Darent Valley Hospital | Dr | Anjana |  | Ray-chaudhuri |
| Collaborator | Derriford Hospital | Mr | Thomas |  | Russell |
| Collaborator | Derriford Hospital | Mr | Andrei |  | Tanase |
| Collaborator | Dumfries and Galloway Royal Infirmary | Miss | Jennifer |  | Downs |
| Collaborator | Dumfries and Galloway Royal Infirmary | Mr | Kieran |  | McCormack |
| Collaborator | Dumfries and Galloway Royal Infirmary | Mr | Muneeb |  | Zafar |
| Collaborator | Dumfries and Galloway Royal Infirmary | Mr | Sharukh |  | Sami |
| Collaborator | East Kent Hospitals University Foundation Trust | Dr | Nashiz |  | Inayet |
| Collaborator | East Kent Hospitals University Foundation Trust | Dr | Wei Jian |  | Tan |
| Collaborator | East Kent Hospitals University Foundation Trust | Dr | Amrita |  | Shandakumar |
| Collaborator | East Kent Hospitals University Foundation Trust | Dr | Varu |  | Udayachandran |
| Collaborator | East Kent Hospitals University Foundation Trust | Dr | Shemin |  | Kovammal |
| Collaborator | East Kent Hospitals University Foundation Trust | Dr | Syed |  | Asfandyar |
| Collaborator | Eastbourne District General Hospital | Dr | Arun |  | Jeevagan |
| Collaborator | Eastbourne District General Hospital | Dr | Christine |  | Higgins |
| Collaborator | Eastbourne District General Hospital | Dr | Hamza |  | Abdelrahim |
| Collaborator | Eastbourne District General Hospital | Mrs | Anita |  | Ivimy |
| Collaborator | Eastbourne District General Hospital | Mrs | Trish |  | Easton |
| Collaborator | Eastbourne District General Hospital | Miss | Charlotte |  | Hughes |
| Regional Lead | Forth Valley Royal Hospital | Miss | Alison |  | Bradley |
| Collaborator | Forth Valley Royal Hospital | Mr | Andrew |  | Crumley |
| Collaborator | Forth Valley Royal Hospital | Dr | Katherine |  | Fox |
| Collaborator | Freeman Hospital | Mr | Gourab |  | Sen |
| Collaborator | Freeman Hospital | Ms | Ghazaleh |  | Mohammadi-Zaniani |
| Regional Lead | Freeman Hospital | Mr | John |  | Moir |
| Collaborator | Freeman Hospital | Mr | Rohan |  | Thakkar |
| Collaborator | Freeman Hospital | Dr | Samuel |  | Moulding |
| Collaborator | Frimley Health NHS FT - Frimley Park | Mr | Darmarajah |  | Veeramootoo |
| Collaborator | Frimley Health NHS FT - Frimley Park | Dr | Adam |  | Kedzierski |
| Collaborator | Frimley Health NHS FT - Frimley Park | Dr | Meghan |  | Coyle |
| Collaborator | Frimley Health NHS FT - Wexham Park | Mr | Kumar |  | Samraj |
| Collaborator | Frimley Health NHS FT - Wexham Park | Mr | Waseem |  | Hameed |
| Collaborator | Frimley Health NHS FT - Wexham Park | Miss | Marriam |  | Ahmed |
| Collaborator | Furness General Hospital | Miss | Panna |  | Patel |
| Collaborator | Furness General Hospital | Mr | Raunaq |  | Chhabra |
| Collaborator | Furness General Hospital | Mr | Siddartha |  | Handa |
| Collaborator | Furness General Hospital | Mr | Shehzad |  | Ahmed |
| Collaborator | Glasgow Royal Infirmary | Dr | Alex |  | Grayston |
| Collaborator | Glasgow Royal Infirmary | Ms | Catherine |  | McCollum |
| Collaborator | Glasgow Royal Infirmary | Mr | David |  | Holroyd |
| Collaborator | Glasgow Royal Infirmary | Mr | Nigel |  | Jamieson |
| Collaborator | Gloucestershire Royal Hospital | Mr | Mark |  | Vipond |
| Collaborator | Gloucestershire Royal Hospital | Mr | Matt |  | Doe |
| Collaborator | Gloucestershire Royal Hospital | Mr | Michael |  | Jones |
| Collaborator | Hammersmith Hospital | Professor | Long |  | Jiao |
| Collaborator | Hammersmith Hospital | Ms | Tamara |  | Gall |
| Collaborator | Hereford County Hospital | Mr | Simon |  | Fisher |
| Collaborator | Hereford County Hospital | Mr | Shahin |  | Hajibandeh |
| Collaborator | Hinchingbrooke Hospital | Mr | Filippo |  | Di Franco |
| Collaborator | Hinchingbrooke Hospital | Dr | Syed |  | Shaukat |
| Collaborator | Hinchingbrooke Hospital | Miss | Julie |  | Ingmire |
| Collaborator | Hinchingbrooke Hospital | Dr | Saima |  | Azam |
| Collaborator | Hinchingbrooke Hospital | Mr | Yogeshkumar |  | Malam |
| Collaborator | Homerton Hospital | Dr | Constantinos |  | Parisinos |
| Collaborator | Homerton Hospital | Dr | Georgina |  | Jackson |
| Collaborator | Homerton Hospital | Dr | Iain |  | Ewing |
| Collaborator | Homerton Hospital | Mr | Thomas |  | Chase |
| Collaborator | Homerton Hospital | Dr | Thomas |  | Ngan |
| Collaborator | Homerton Hospital | Dr | Wafaa |  | Hajee-Adam |
| Collaborator | Huddersfield Royal Infirmary CHFT | Dr | Ashwin |  | Verma |
| Collaborator | Huddersfield Royal Infirmary CHFT | Ms | Melissa |  | Prior-Ong |
| Collaborator | Huddersfield Royal Infirmary CHFT | Ms | Claire |  | McDonald |
| Collaborator | Hull University Teaching Hospitals NHS Trust | Dr | Alasdair |  | Findlay |
| Collaborator | Hull University Teaching Hospitals NHS Trust | Dr | Bethany |  | Scutt |
| Collaborator | Hull University Teaching Hospitals NHS Trust | Dr | Brianda |  | Ripoll |
| Collaborator | Hull University Teaching Hospitals NHS Trust | Mr | Hussamuddin |  | Adwan |
| Collaborator | Hull University Teaching Hospitals NHS Trust | Dr | Jordan |  | Green |
| Collaborator | Hull University Teaching Hospitals NHS Trust | Mr | Pavlos |  | Lykoudis |
| Collaborator | Inverclyde Royal Hospital | Mr | Jasim |  | Amin |
| Collaborator | Inverclyde Royal Hospital | Ms | Mei ying |  | Chin |
| Collaborator | Inverclyde Royal Hospital | Dr | Conor |  | Smith |
| Collaborator | Inverclyde Royal Hospital | Dr | Tharsika |  | Kuganesan |
| Collaborator | Ipswich Hospital | Ms | Gemma |  | Aldous |
| Collaborator | Ipswich Hospital | Dr | Hemant |  | Laxaman |
| Collaborator | Ipswich Hospital | Dr | Usama |  | Aslam |
| Collaborator | James Paget Hospital | Mrs | Joanne |  | Giles |
| Collaborator | James Paget Hospital | Dr | Matthew |  | Williams |
| Collaborator | James Paget Hospital | Mrs | Tracey |  | Noakes |
| Collaborator | James Paget Hospital | Dr | Zeshan |  | Choudhry |
| Collaborator | Kettering General Hospital | Mrs | Amy |  | Shroll |
| Collaborator | Kettering General Hospital | Dr | Muthi |  | Kasimanickam |
| Collaborator | Kettering General Hospital | Ms | Monica |  | Palmer |
| Collaborator | University Hospitals of Leicester NHS Trust | Professor | Giuseppe |  | Garcea |
| Regional Lead | University Hospitals of Leicester NHS Trust | Dr | Amar |  | Kourdouli |
| Collaborator | University Hospitals of Leicester NHS Trust | Dr | Mariuca |  | Popa |
| Collaborator | Lister Hospital | Mr | Paul |  | Jose |
| Collaborator | Lister Hospital | Dr | Alexander |  | West |
| Collaborator | Lister Hospital | Dr | Marios |  | Erotocritou |
| Collaborator | Liverpool Universities Hospital - Royal Site | Dr | Elizabeth |  | O'Connell |
| Collaborator | Liverpool Universities Hospital - Royal Site | Mr | Kulbir |  | Mann |
| Collaborator | Liverpool Universities Hospital - Royal Site | Mr | Leonard |  | Quinn |
| Collaborator | Luton and Dunstable University Hospital | Mr | Roy |  | Gurprashad |
| Collaborator | Luton and Dunstable University Hospital | Mr | Douglas |  | Whitelaw |
| Regional Lead | Luton and Dunstable University Hospital | Miss | Chelise |  | Currow |
| Collaborator | Luton and Dunstable University Hospital | Dr | Edward |  | Saxton |
| Collaborator | Luton and Dunstable University Hospital | Dr | Harkiran |  | Sagoo |
| Collaborator | Luton and Dunstable University Hospital | Miss | Tolu |  | Ekong |
| Collaborator | Maidstone and Tunbridge Wells NHS Trust | Dr | Archil |  | Tsirekidze |
| Collaborator | Maidstone and Tunbridge Wells NHS Trust | Dr | Justin |  | Waters |
| Collaborator | Maidstone and Tunbridge Wells NHS Trust | Dr | Veena |  | Ramachandran |
| Collaborator | Manchester Royal Infirmary | Professor | Ajith | K | Siriwardena |
| Collaborator | Manchester Royal Infirmary | Dr | Joe |  | Geraghty |
| Collaborator | Manchester Royal Infirmary | Dr | Matthew |  | Goldsworthy |
| Collaborator | Manchester Royal Infirmary | Mr | Nicola |  | de Liguori-Carino |
| Collaborator | Morriston Hospital Swansea | Professor | Bilal |  | Al-Sarireh |
| Collaborator | Morriston Hospital Swansea | Mr | Christopher |  | Brown |
| Collaborator | Morriston Hospital Swansea | Mr | Guy |  | Shingler |
| Collaborator | Morriston Hospital Swansea | Dr | Jennifer |  | Hayes |
| Collaborator | NHS Grampian | Mr | Bassam |  | Alkari |
| Collaborator | NHS Grampian | Professor | Irfan |  | Ahmed |
| Collaborator | NHS Grampian | Mr | James |  | Milburn |
| Collaborator | NHS Grampian | Ms | Opeyemi |  | Sogaolu |
| Collaborator | NHS Grampian | Mr | Mohammad |  | Hassan |
| Collaborator | Norfolk and Norwich University Hospital | Miss | Laura |  | Gale |
| Collaborator | Norfolk and Norwich University Hospital | Dr | Sathis |  | Mogan |
| Collaborator | North Middlesex University Hospital NHS Trust | Dr | Andrew |  | Millar |
| Collaborator | North Middlesex University Hospital NHS Trust | Dr | Aria |  | Khani |
| Collaborator | North Middlesex University Hospital NHS Trust | Dr | Debasis |  | Majumdar |
| Collaborator | North Middlesex University Hospital NHS Trust | Dr | Samuel |  | Morris |
| Collaborator | North Tees and Hartlepool NHS Trust | Dr | Khalid |  | Bashir |
| Collaborator | North Tees and Hartlepool NHS Trust | Ms | Linda |  | Butler |
| Collaborator | North Tees and Hartlepool NHS Trust | Dr | Mohamed |  | Elzubier |
| Collaborator | North Tees and Hartlepool NHS Trust | Mrs | Natalie |  | Robson |
| Collaborator | North Tees and Hartlepool NHS Trust | Mrs | Stephanie |  | Burns |
| Collaborator | North Tees and Hartlepool NHS Trust | Dr | Vikramjit |  | Mitra |
| Collaborator | Northampton General Hospital | Mr | Guy |  | Finch |
| Collaborator | Northampton General Hospital | Ms | Hannah |  | Byrne |
| Collaborator | Northampton General Hospital | Dr | Xavier |  | Fung |
| Collaborator | Northampton General Hospital | Dr | Robert |  | Bailey |
| Collaborator | Northampton General Hospital | Dr | Syed |  | Rizvi |
| Collaborator | Northampton General Hospital | Dr | Shahriar |  | Reza |
| Collaborator | Northumbria Healthcare Trust | Miss | Emily |  | Thompson |
| Collaborator | Northumbria Healthcare Trust | Miss | Sarah |  | Robinson |
| Collaborator | Northumbria Healthcare Trust | Dr | Maurice |  | Samake |
| Collaborator | Nottingham City Hospital | Mr | Glen |  | Irving |
| Collaborator | Nottingham City Hospital | Dr | Suresh Vasan |  | Venkatachalapathy |
| Collaborator | Nottingham City Hospital | Dr | Nasir |  | Javed |
| Collaborator | Nottingham City Hospital | Dr | Michael |  | Ho |
| Collaborator | Nottingham City Hospital | Dr | Syed |  | Karim |
| Collaborator | Peterborough City Hospital | Ms | Seok Ling |  | Ong |
| Collaborator | Peterborough City Hospital | Ms | Sophie |  | Tucker |
| Collaborator | Peterborough City Hospital | Ms | Panchali |  | Sarmah |
| Collaborator | Poole Hospital | Dr | Melissa |  | Zhao |
| Collaborator | Poole Hospital | Dr | Nicholas |  | Sharer |
| Collaborator | Poole Hospital | Dr | William |  | Knibbs |
| Collaborator | Princess Alexandra Hospital | Dr | Lushen |  | Pillay |
| Collaborator | Princess Alexandra Hospital | Ms | Polly |  | Rogers |
| Collaborator | Princess Alexandra Hospital | Dr | Rosemary |  | Phillips |
| Regional Lead | Queen Alexandra Hospital | Dr | Benjamin |  | Giles |
| Collaborator | Queen Alexandra Hospital | Dr | Megan |  | Rowley |
| Collaborator | Queen Alexandra Hospital | Dr | Richard |  | Aspinall |
| Collaborator | Queen Elizabeth Hospital Birmingham | Miss | Sarah |  | Powell-Brett |
| Collaborator | Queen Elizabeth Hospital Birmingham | Mr | Ahmed |  | Almonib |
| Collaborator | Queen Elizabeth Hospital Birmingham | Mr | Danylo |  | Yershov |
| Regional Lead | Queen Elizabeth Hospital Birmingham | Mr | Fahad |  | Mahmood |
| Collaborator | Queen Elizabeth Hospital Birmingham | Mr | Kunal |  | Joshi |
| Collaborator | Queen's Hospital Burton | Dr | Altaf |  | Palejwala |
| Collaborator | Queen's Hospital Burton | Mrs | Sarah |  | Johnson |
| Collaborator | Queen's Hospital Burton | Dr | Joao |  | Pinheiro |
| Collaborator | Queen's Hospital Romford | Dr | Amy |  | Ward |
| Regional Lead | Queen's Hospital Romford | Dr | Cavitha |  | Vivekananthan |
| Collaborator | Royal Blackburn Hospital | Ms | Ambareen |  | Kausar |
| Collaborator | Royal Blackburn Hospital | Mr | Joseph |  | Mcaleer |
| Collaborator | Royal Bournemouth Hospital | Mr | Nick |  | Davies |
| Collaborator | Royal Bournemouth Hospital | Miss | Agata |  | Majkowska |
| Collaborator | Royal Bournemouth Hospital | Dr | Phoebe |  | Wilson |
| Collaborator | Royal Derby Hospital | Mr | Imran |  | Bhatti |
| Collaborator | Royal Derby Hospital | Dr | Christiana |  | Fabelurin |
| Collaborator | Royal Derby Hospital | Mr | Nanda |  | Bandlamudi |
| Collaborator | Royal Devon and Exeter Hospital | Dr | Elizabeth |  | Toy |
| Collaborator | Royal Devon and Exeter Hospital | Dr | Tim |  | Norris |
| Collaborator | Royal Free Hospital London | Mr | Stephanos |  | Pericleous |
| Collaborator | Royal Free Hospital London | Professor | Giuseppe |  | Fusai |
| Collaborator | Royal Free Hospital London | Dr | Roopinder |  | Gillmore |
| Collaborator | Royal Free Hospital London | Dr | Alvin |  | Lee |
| Collaborator | Royal Glamorgan Hospital | Mr | Tim |  | Havard |
| Collaborator | Royal Glamorgan Hospital | Mr | Benjamin |  | Tinsley |
| Collaborator | Royal Gwent Hospital | Dr | Luke |  | Taylor |
| Collaborator | Royal Gwent Hospital | Ms | Tamsin |  | Boyce |
| Collaborator | Royal Lancaster Infirmary | Mrs | Emma |  | Davies |
| Collaborator | Royal Lancaster Infirmary | Dr | Lysia |  | Richmond |
| Collaborator | Royal Lancaster Infirmary | Miss | Rebecca |  | Varley |
| Collaborator | Royal Lancaster Infirmary | Mr | Nader |  | Adel |
| Collaborator | Royal Lancaster Infirmary | Dr | Utitofon |  | Ekpenyong |
| Collaborator | Royal Lancaster Infirmary | Dr | Zain |  | Sultan |
| Collaborator | Royal Lancaster Infirmary | Dr | Hind |  | Rassam |
| Collaborator | Royal London Hospital | Dr | David |  | Propper |
| Collaborator | Royal London Hospital | Professor | Hemant |  | Kocher |
| Collaborator | Royal London Hospital | Miss | Pallavi |  | Arya |
| Collaborator | Royal London Hospital | Dr | Sarah |  | Slater |
| Collaborator | Royal Sussex County Hospital | Dr | Mandeep |  | Kaur |
| Collaborator | Royal Sussex County Hospital | Dr | Adel |  | Hamed |
| Collaborator | Royal Sussex County Hospital | Mr | Shameen |  | Jaunoo |
| Collaborator | Royal Sussex County Hospital | Mr | Tarek |  | El-housseri |
| Collaborator | Royal Victoria Hospital | Mr | Mark |  | Taylor |
| Collaborator | Royal Victoria Hospital | Ms | Claire |  | Jones |
| Collaborator | Royal Victoria Hospital | Mr | Daniel |  | Kane |
| Collaborator | Royal Victoria Hospital | Mr | Stephen |  | McCain |
| Collaborator | Royal Victoria Hospital | Dr | Lauren |  | Laverty |
| Collaborator | Russells Hall Hospital | Dr | Ameer |  | Mustafa |
| Collaborator | Russells Hall Hospital | Mr | Chaminda |  | Sellahewa |
| Collaborator | Sandwell General Hospital | Mr | Osama |  | Zaman |
| Collaborator | Sandwell General Hospital | Mr | Yogesh |  | Kumar |
| Collaborator | Southampton General Hospital | Mr | Ali |  | Arshad |
| Collaborator | Southampton General Hospital | Miss | Claire |  | Stevens |
| Collaborator | Southampton General Hospital | Miss | Lulu |  | Tanno |
| Collaborator | Southend University Hospital | Dr | Daniel |  | Waite |
| Collaborator | Southend University Hospital | Dr | Ioannis |  | Koumoutsos |
| Collaborator | Southend University Hospital | Mrs | Sophie |  | Laverick |
| Collaborator | Southend University Hospital | Mrs | Ann |  | Beluso |
| Collaborator | Southend University Hospital | Mrs | Tracey |  | Turner |
| Collaborator | Southend University Hospital | Dr | David |  | Tsang |
| Collaborator | St George's Hospital | Mr | Dimitrios |  | Tsironis |
| Collaborator | St George's Hospital | Dr | Sophie |  | Barker |
| Regional Lead | St George's Hospital | Mr | Mohamed |  | Abouelazayem |
| Collaborator | St George's Hospital | Miss | Svetlana |  | Ciocarlan |
| Collaborator | St George's Hospital | Miss | Raluca |  | Belchita |
| Collaborator | St George's Hospital | Dr | Yooyun |  | Chung |
| Collaborator | St James's University Hospital Leeds | Mr | Alistair |  | Young |
| Collaborator | St James's University Hospital Leeds | Mr | Andrew |  | Smith |
| Collaborator | St James's University Hospital Leeds | Mr | Nikhil |  | Suresh |
| Collaborator | St James's University Hospital Leeds | Mr | Tom |  | Pike |
| Collaborator | St James's University Hospital Leeds | Mr | Usman |  | Khokar |
| Collaborator | The Royal Wolverhampton NHS Trust | Dr | Shyam |  | Menon |
| Collaborator | The Royal Wolverhampton NHS Trust | Dr | Muhammad Raheel | | Anjum |
| Collaborator | The Royal Wolverhampton NHS Trust | Dr | Reuben |  | Kurien |
| Collaborator | Torbay and South Devon NHS Foundation Trust | Dr | Ben |  | Keatley |
| Collaborator | Torbay and South Devon NHS Foundation Trust | Dr | Luke |  | Summers |
| Collaborator | Torbay and South Devon NHS Foundation Trust | Dr | Rhys |  | Williams |
| Collaborator | Torbay and South Devon NHS Foundation Trust | Dr | William |  | O'Rourke |
| Collaborator | University College London Hospitals | Professor | Stephen |  | Pereira |
| Collaborator | University College London Hospitals | Dr | Harry |  | Martin |
| Collaborator | University Hospital North Midlands NHS Trust | Mr | Tejinderjit |  | Athwal |
| Collaborator | University Hospital North Midlands NHS Trust | Mr | Nader |  | Ghassemi |
| Collaborator | University Hospital North Midlands NHS Trust | Mr | Yanish |  | Poolovadoo |
| Collaborator | University Hospital North Midlands NHS Trust | Mr | Joseph |  | Meilak |
| Collaborator | University Hospital North Midlands NHS Trust | Dr | Alyssa |  | Ralph |
| Collaborator | University Hospital North Midlands NHS Trust | Dr | Amal |  | Boulbadaoui |
| Collaborator | University Hospital of North Durham | Dr | Deepak |  | Kejariwal |
| Collaborator | University Hospital of North Durham | Mrs | Donna |  | Thomas |
| Collaborator | University Hospital of Wales | Mr | David |  | O'Reilly |
| Collaborator | University Hospital of Wales | Mr | Matthew |  | Mortimer |
| Regional Lead | University Hospital of Wales | Mr | Nicholas |  | Mowbray |
| Collaborator | University Hospital of Wales | Mr | Dylan |  | Jones |
| Meta-Coordinator | University of Birmingham | Mr | Thomas |  | Thorne |
| Meta-Coordinator | University of Birmingham | Mr | Jacek |  | Parylo |
| Meta-Coordinator | University of Birmingham | Mr | Samuel |  | Hodgson |
| Meta-Coordinator | University of Birmingham | Mr | Daniel |  | Smith |
| Meta-Coordinator | University of Birmingham | Mr | Oscar |  | Hargreaves |
| Meta-Coordinator | University of Birmingham | Mr | Thomas |  | Binnersly |
| Meta-Coordinator | University of Birmingham | Mr | Samuel |  | Brown |
| Meta-Coordinator | University of Birmingham | Miss | Radhika |  | Acharya |
| Meta-Coordinator | University of Birmingham | Miss | Rebecca |  | Everitt |
| Meta-Coordinator | University of Birmingham | Miss | Madeleine |  | Perrett |
| Collaborator | University Plymouth NHS Trust | Mr | Andrei |  | Tanase |
| Collaborator | University Plymouth NHS Trust | Mr | Thomas |  | Russell |
| Collaborator | University Plymouth NHS Trust | Mr | Somaiah |  | Aroori |
| Collaborator | Victoria Hospital Kirkcaldy | Ms | Danielle |  | Clyde |
| Collaborator | Victoria Hospital Kirkcaldy | Ms | Morag |  | Mclellan |
| Collaborator | Victoria Hospital Kirkcaldy | Mr | Syed |  | Naqvi |
| Collaborator | Walsall Manor Hospital | Miss | Sian |  | Abbott |
| Collaborator | Walsall Manor Hospital | Miss | Alexia |  | Farrugia |
| Collaborator | Walsall Manor Hospital | Dr | Robyn |  | Marsh |
| Collaborator | Walsall Manor Hospital | Dr | Oluwafemi |  | Osunlusi |
| Collaborator | Warrington and Halton Teaching Hospitals NHS Foundation Trust | Dr | Chew |  | Tan |
| Collaborator | Warrington and Halton Teaching Hospitals NHS Foundation Trust | Dr | Jaiganesh |  | Mohan |
| Collaborator | Warrington and Halton Teaching Hospitals NHS Foundation Trust | Dr | Lakshmi Deepa |  | Vandadi |
| Collaborator | West Middlesex Hospital | Dr | Georgina |  | Chadwick |
| Collaborator | West Suffolk Hospital | Mr | Amitabh |  | Mishra |
| Collaborator | West Suffolk Hospital | Mr | Akshay |  | Bavikatte |
| Collaborator | Whittington Hospital NHS Trust, London | Mr | Chetan |  | Parmar |
| Regional Lead | Whittington Hospital NHS Trust, London | Ms | Roxanna |  | Zakeri |
| Collaborator | Whittington Hospital NHS Trust, London | Dr | Michael |  | Hanna |
| Collaborator | Worcestershire Royal Hospital | Mr | Moustafa |  | Mourad |
| Collaborator | Worcestershire Royal Hospital | Mr | Ahmed |  | Elmaradny |
| Collaborator | Wrexham Maelor | Mr | Duncan |  | Stewart |
| Collaborator | Wrexham Maelor | Miss | Corina |  | Lavelle |
| Collaborator | Ysbyty Gwynedd (Bangor) | Dr | Khaled |  | Radwan |
| Collaborator | Ysbyty Gwynedd (Bangor) | Dr | Mustafa |  | Gherghab |
| Collaborator | Ysbyty Gwynedd (Bangor) | Dr | Jaber |  | Gasem |
| Collaborator | Ysbyty Gwynedd (Bangor) | Dr | Areeb |  | Khan |
| Collaborator | Ysbyty Gwynedd (Bangor) | Dr | Jonathan |  | Sutton |
| Collaborator | Ysbyty Gwynedd (Bangor) | Ms | Vicky |  | Jones |
| BiCOPS Research Manager | Birmingham Centre for Observational and Prospective Studies (BiCOPS) | Mr | Michael |  | Walters |
| BiCOPS Research Facilitator | Birmingham Centre for Observational and Prospective Studies (BiCOPS) | Mr | Terry |  | Hughes |
| BiCOPS Research Manager | Birmingham Centre for Observational and Prospective Studies (BiCOPS) | Ms | Rita |  | Perry |
